# Combining Contextualized Embeddings and Prior Knowledge for Clinical Named Entity Recognition: Evaluation Study

**DOI:** 10.2196/14850

**Published:** 2019-11-13

**Authors:** Min Jiang, Todd Sanger, Xiong Liu

**Affiliations:** 1 Eli Lilly and Company Indianapolis, IN United States

**Keywords:** natural language processing, named entity recognition, deep learning, contextualized word embedding, semantic embedding, prior knowledge

## Abstract

**Background:**

Named entity recognition (NER) is a key step in clinical natural language processing (NLP). Traditionally, rule-based systems leverage prior knowledge to define rules to identify named entities. Recently, deep learning–based NER systems have become more and more popular. Contextualized word embedding, as a new type of representation of the word, has been proposed to dynamically capture word sense using context information and has proven successful in many deep learning–based systems in either general domain or medical domain. However, there are very few studies that investigate the effects of combining multiple contextualized embeddings and prior knowledge on the clinical NER task.

**Objective:**

This study aims to improve the performance of NER in clinical text by combining multiple contextual embeddings and prior knowledge.

**Methods:**

In this study, we investigate the effects of combining multiple contextualized word embeddings with classic word embedding in deep neural networks to predict named entities in clinical text. We also investigate whether using a semantic lexicon could further improve the performance of the clinical NER system.

**Results:**

By combining contextualized embeddings such as ELMo and Flair, our system achieves the F-1 score of 87.30% when only training based on a portion of the 2010 Informatics for Integrating Biology and the Bedside NER task dataset. After incorporating the medical lexicon into the word embedding, the F-1 score was further increased to 87.44%. Another finding was that our system still could achieve an F-1 score of 85.36% when the size of the training data was reduced to 40%.

**Conclusions:**

Combined contextualized embedding could be beneficial for the clinical NER task. Moreover, the semantic lexicon could be used to further improve the performance of the clinical NER system.

## Introduction

### History of Clinical Named Entity Recognition

Clinical named entity recognition (NER), an important clinical natural language processing (NLP) task, has been explored for several decades. In the early stage, most NER systems leverage rules and dictionaries to represent linguistic features and domain knowledge to identify clinical entities, such as MedLEE [1], SymText/MPlus [2,3], MetaMap [4], KnowledgeMap [5], cTAKES [6], and HiTEX [7]. To promote the development of machine learning–based system, many publicly available corpora have been developed by organizers of some clinical NLP challenges such as the Informatics for Integrating Biology and the Bedside (i2b2) 2009 [8], 2010 [9-13], 2012 [14-18], 2014 [19-23], ShARe/CLEF eHealth Evaluation Lab 2013 dataset [24], and Semantic Evaluation 2014 task 7 [25], 2015 task 6 [26], 2015 task 14 [27], and 2016 task 12 [28] datasets. Many machine learning–based clinical NER systems have been proposed, and they greatly improved performance compared with the early rule-based systems [13,29,30]. Most systems are implemented based on two types of supervised machine learning algorithms: (1) classification algorithms such as support vector machines (SVMs) and (2) sequence labeling algorithms such as conditional random fields (CRFs), hidden Markov models (HMMs), and structural support vector machines (SSVMs). Among all of the algorithms, CRFs play the leading roles due to the advantage of the sequence labeling algorithms over classification algorithms in considering context information when making the prediction; CRFs, as one type of discriminative model, tend to achieve better performance for the same source of testing data compared with generative model-based algorithms such as HMMs. Even though CRFs have achieved a huge success in the clinical NER area, they have some obvious limitations: CRF-based systems lie in manually crafted features, which are time consuming, and their ability to capture context in a large window is limited.

### Deep Neural Network–Based Named Entity Recognition Algorithms

In recent years, deep neural network–based NER algorithms have been extensively studied, and many deep learning–based clinical NER systems have been proposed. They have an obvious advantage over traditional machine learning algorithms since they do not require feature engineering, which is the most difficult part of designing machine learning–based systems. They also improve the ability to leverage the context information. Initially, word embedding [31] is proposed as a method to represent the word in a continuous way to better support neural network structure. Then several new neural network structures including recurrent neural networks (RNNs) and long short-term memory (LSTM) [32] have been introduced to better represent sequence-based input and overcome long-term dependency issues. Recently, contextual word representations generated from pretrained bidirectional language models (biLMs) have been shown to significantly improve the performance of state-of-the-art NER systems [33].

**Figure 1:**
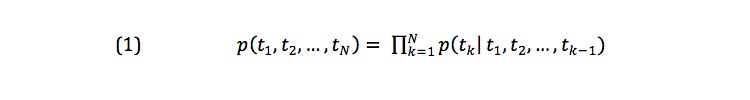
Sequence probability in bidirectional language models.

In biLMs, the language model (LM) can be described as: given a sequence of N tokens, (*t_1_*, *t_2_*, ..., *t_N_*), the probability of token *t_k_* can be calculated given the history (*t_1_*, ..., *t_k–1_*), and the sequence probability can be computed as seen in Figure 1.

**Figure 2:**
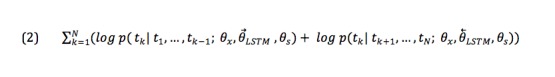
Log-likelihood of the forward and backward directions language models.

Recent neural LMs usually include one layer of token input, which is represented by word embedding or a CNN over characters, followed by L layers of forward LSTMs. On the top layer, the SoftMax layer is added to generate a prediction score for the next token [33]. The biLM combines two such neural LMs: the forward LM and backward LM; the backward LM is similar to the forward LM, except it runs over the reverse sequence. As a whole, the biLM tries to maximize the log-likelihood of the forward and backward directions as seen in Figure 2.

Where θ_x_ represents the token representation layer, θ_s_ represents the Softmax layer, and 
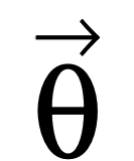
*_LSTM_* and 
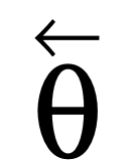
*_LSTM_* represent the forward and backward directions of the LSTM layer.

In 2017, Peters et al [34] introduced a sequence tagger called TagLM that combines pretrained word embeddings and biLM embeddings as the representation of the word to improve the performance of the NER system. Since the output of each layer of the biLM represents a different type of contextual information [35], Peters et al [33] proposed another embedding, a deep contexualized word representation, ELMo, by concatenating all the biLM layer outputs into the biLM embedding with a weighted average pooling operation. The ELMo embedding adds CNN and highway networks over the character for each token as the input. ELMo has been proven to enhance the performance of different NLP tasks such as semantic role labeling and question answering [33].

**Figure 3:**
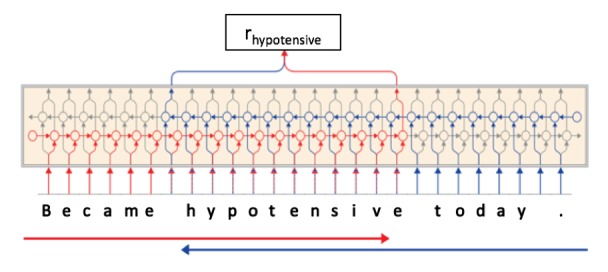
Architecture of extracting a contextual string embedding.

Similar to Peters’ ELMo, Akbik et al [36] introduced contextual string embeddings for sequence labeling, which leverages neural character-level language modeling to generate a contextualized embedding for each word input within a sentence. The principle of the character-level LM is that it is the same as biLMs except that it runs on the sequences of characters instead of tokens. Figure 3 shows the architecture of extracting a contextual string embedding for the word “hypotensive” in a sentence. We can see that instead of generating a fixed representation of the embedding for each word, the embedding of each token is composed of pretrained character embeddings from surrounding text, meaning the same token has dynamic representation depending on its context.

### Deep Neural Network–Based Clinical Named Entity Recognition Systems

In the clinical domain, researchers investigated the performance of clinical NER tasks on various types of deep neural network structures. In 2015, researchers showed it is beneficial to use the large clinical corpus to generate word embeddings for clinical NER systems, and they comparatively investigated the different ways of generating word embeddings in the clinical domain [37]. In 2017, Wu et al [38] produced state-of-the-art results on the i2b2 2010 NER task dataset by employing the LSTM-CRF structure. Liu et al [39] investigated the effects of two types of character word embeddings on LSTM-based systems on multiple i2b2/Veterans Administration (VA) NER task datasets. In 2018, Zhu et al [40] employed a contextualized LM embedding on clinical data and boosted the state-of-the-art performance by 3.4% on the i2b2/VA 2010 NER dataset. The above studies show that, with the development of methods in text representation learning, especially contextual word embedding, more and more hidden knowledge can be learned from a large unannotated clinical corpus, which is beneficial for clinical NER tasks. According to the study by Peters et al [35], contextual word representations derived from pretrained biLMs can learn different levels of information that vary with the depth of the network, from local syntactic information to long-range dependent semantic information. Even without leveraging traditional domain knowledge such as lexicon and ontology, deep learning–based NER systems can achieve better performance than traditional machine learning–based systems.

Besides using pretrained representation from large unlabeled corpora, researchers started to integrate prior knowledge into deep learning frameworks to improve the performance of the NER system. For example, in the general domain, Yu and Dredze [41] created a semantic word embedding based on WordNet and evaluated the performance on language modeling, semantic similarity, and human judgment prediction. In another example, Weston et al [42] leveraged a CNN to generate a semantic embedding based on hashtags to improve the performance of the document recommendation task. In the clinical domain, Wu et al [43] compared two types of methods to inject medical knowledge into deep learning–based clinical NER solutions and found that the RNN-based system combining medical knowledge as embeddings achieved the best performance on the i2b2 2010 dataset. In 2019, Wang et al [44] explored two different architectures that extend the bidirectional LSTM (biLSTM) neural network and five different feature representation schemes to incorporate the medical dictionaries. In addition, other studies also use prior knowledge to generate embeddings [45-49].

To date, no detailed analysis has been published to investigate the value of combining different types of word embeddings and prior knowledge for clinical NER. In this study, we made the following contributions: (1) we proposed an innovative method to combine two types of contextualized embeddings to study their effects on the clinical NLP challenge dataset, (2) we incorporated prior knowledge from semantic resources such as medical lexicon to evaluate if it could further improve the performance of the clinical NER system, and (3) we conducted a thorough evaluation on our models with different sizes of data to gain knowledge on how much data are needed to train a high-performance clinical NER system.

## Methods

### Datasets

For this study, we used two datasets, the 2010 i2b2/VA concept extraction track dataset and the Medical Information Mart for Intensive Care III (MIMIC-III) corpus. The 2010 i2b2/VA challenge dataset is annotated with named entities, while the MIMIC-III corpus is unannotated data.

#### 2010 i2b2/VA Concept Extraction Track Dataset

The goal of the 2010 i2b2/VA concept extraction task is to identify three types of clinical named entities including problem, treatment, and test from clinical notes. The original dataset includes 349 notes in the training set and 477 notes in the testing set, which include discharge summaries and progress notes from three institutions: Partners HealthCare, Beth Israel Deaconess Medical Center, and University of Pittsburgh Medical Center. Since the University of Pittsburgh Medical Center’s data have been removed from the original data set, the portion of discharge summaries that is available contains 170 notes for training and 256 for testing. In total, the training set contains 16,523 concepts including 7073 problems, 4844 treatments, and 4606 tests. The test set contains 31,161 concepts including 12,592 problems, 9344 treatments, and 9225 tests.

#### Medical Information Mart for Intensive Care III Corpus

The MIMIC-III corpus [50] is from MIMIC-III database, which is a large, freely available de-identified health-related dataset that integrates de-identified, comprehensive clinical data of patients admitted to the Beth Israel Deaconess Medical Center in Boston, Massachusetts.

The dataset comprises 2,083,180 notes from 15 different note types including “rehab services,” “case management,” “general,” “discharge summary,” “consult,” “radiology,” “electrocardiography,” “nutrition,” “social work,” “pharmacy,” “echocardiography,” “physician,” “nursing,” “nursing/other,” and “respiratory.”

### Embedding Generation

In order to fit our text input into the deep neural network structure, we generated three types of embeddings: classic word embeddings, (2) contextualized LM–based word embeddings, and semantic word embeddings.

#### Training Classic Word Embeddings

We generated two types of word embeddings based on the MIMIC-III corpus and a medical lexicon: MIMIC-III corpus-based embeddings and tagged MIMIC-III corpus-based embeddings. We adopted the Word2Vec implementation database from Github [51] to train word embeddings based on the MIMIC-III corpus. We used a continuous bag-of-words architecture with negative sampling. In accordance with the results from the study by Xu et al [52], we set the dimension of embedding as 50.

#### Training Contextual Language Model–Based Embeddings

Besides the word embeddings, we employed two recently proposed methods to generate contextual LM-based embeddings: ELMo embeddings and (2) contextual string embeddings for sequence labeling (Flair).

### Training ELMo Embeddings

We followed the method introduced by Zhu et al [40] that uses a partial MIMIC-III corpus combined with a certain portion of Wikipedia pages as a training corpus to train the ELMo contextual LM in the clinical domain. In more detail, it combines discharge summaries and radiology reports from the MIMIC-III corpus and all the Wikipedia pages with titles that are items from the Systematized Nomenclature of Medicine–Clinical Terms. Such a corpus is trained on a deep neural network that contains a character-based CNN embedding layer followed by a two-layer biLSTM. Details have been published elsewhere [40].

### Training Contextual String Embeddings for Sequence Labeling

Akbik et al [36] proposed a new method to generate a neural character-level LM. The paper shows the state-of-the-art performance on the Conference on Computational Natural Language Learning 2003 NER task dataset. The LM for the general domain is publicly accessible. The author also integrates all the codes into an NLP framework called Flair. It achieved great success on the data in the general domain. However, according to the research by Friedman et al [53], clinical language has unique linguistic characteristics compared with general English, which make models generated from the public domain poorly adaptable to clinical narratives. It is demanding to train the LM on the clinical corpus to better support the clinical NER task. For training corpus preparation, we first did sentence segmentation on the entire corpus, then we randomly selected 1500 sentences as the testing set and another 1500 sentences for the validation set. The remaining part serves as the training set. For the hyperparameters, we kept the default setting: learning rate as 20.0, batch size as 32, anneal factor as 0.25, patience as 10, clip as 0.25, and hidden size as 1024.

### Training Semantic Word Embeddings

**Figure 4:**
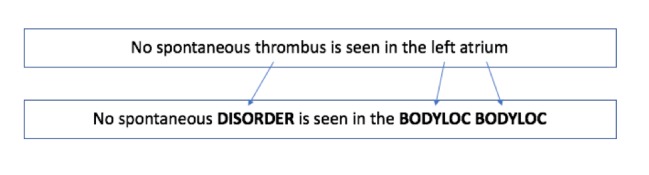
One example of converting the sentence into the tagged sentence.

Injecting domain knowledge into the deep learning model is a potential way to further improve the performance of the NER system. According to the results by Wu et al [43], combining medical knowledge into the embedding outperforms the method of representing it as a one-hot vector. Therefore, we similarly created the embedding to represent medical lexicon and fed it into the deep learning framework in our study. More specifically, we initially generated a lexicon dictionary based on a subset of semantic categories in the Unified Medical Language System. We then identified all the lexicon occurrences in the corpus using the dictionary and replaced them with semantic categories. Figure 4 shows an example of the conversion. In the example sentence of “No spontaneous thrombus is seen in the left atrium,” “thrombus” is replaced with the tag “DISORDER” and “left atrium” is replaced with two “BODYLOC” tags. In this way, we can integrate semantic information into the word embeddings. For the embedding generation, we use the same setting as in the previous section.

### Deep Neural Network Architecture

After we generated all the embeddings, we started to fit them as the input into our deep neural network for the supervised training stage. Since each type of embedding is generated using one method, meaning each represents different aspects of knowledge from the large corpus, combining them is an obvious solution to potentially further improve the performance, which has also been proven by clinical NER studies [40,43]. Although there are many options to combine multiple embeddings in the deep neural network system such as weighting [54] and ensemble [55], in this study, we adopted the most straightforward way, which is simply concatenating them as the input.

**Figure 5:**
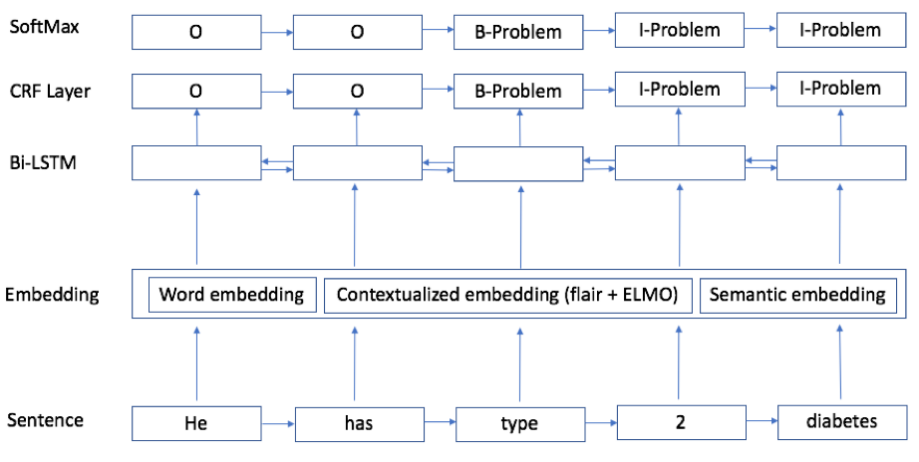
Deep neural network structure with combined embeddings. Bi-LSTM: bidirectional long short-term memory; CRF: conditional random field.

We used the biLSTM-CRF sequence labeling module proposed by Huang et al [56]. Figure 5 shows the architecture of the whole deep neural network structure; the input is the embedding layer, which is concatenated by different types of embeddings as described in the previous section. Before we extracted embeddings for tagged word embedding, we used the same medical lexicon–based tagger to replace the tokens with the semantic tags. All the embedding inputs went through the biLSTM layer to generate forward and backward output, which was used to calculate the probability score by CRF layers. On the top, the prediction was given by a SoftMax layer.

### Training the Deep Neural Network–Based Sequence Tagger

For the implementation, we employed Flair [57], which is a simple framework for NLP tasks including NER and text classification. We used the default hyperparameter setting in Flair, and we used the following configuration: learning rate as 0.1, batch size as 32, dropout probability as 0.5, and maximum epoch as 500. The learning rate annealing method is basically the same as the default: we half the learning rate if the training loss does not fall for the consecutive “patience” number of epochs. We set the patience number to 12 in this study. A TITAN V (NVIDIA Corporation) graphics processing unit was used to train the model. We took about 4 hours to train our model each time.

### Evaluation

In order to get more reliable results, we ran each model three times. For the measurement of each running, we used precision, recall, and F-1 score.

## Results

Table 1 shows the performance of the challenge winner system and different deep neural network systems. We used four benchmarks as our baseline systems, and then we reported the performance of the systems when adding ELMo embeddings, Flair embeddings, and tagged embeddings one at a time. All evaluation scores were based on exact matching. For the baseline systems, the first one is the semi-Markov model, developed by Debruijn et al [13], which reported an F-1 score of 85.23%. The second and third baselines are both based on the LSTM model, and they reported F-1 scores of 85.78% and 85.94%, respectively. The last baseline is the best result for the nonensemble models from Zhu et al [40], which used ELMo embedding. The three baseline systems used the original corpus (training: 349 notes; test: 477 notes), all other systems are based on the existing modified corpus (training: 170 notes; test: 256 notes). To start, we combined word embeddings with ELMo and Flair embeddings, respectively. Both models achieved an F-1 score of 87.01%, which is a little bit higher than what was reported by Zhu et al [40]. After combining word embeddings with ELMo and Flair embeddings, the F-1 score increased to 87.30%. When the word embedding on the tagged corpus was incorporated, the performance was further improved to 87.44% for the F-1 score.

In order to test if the improvement between different results is statistically significant, we conducted a statistical test based on results from bootstrapping. From the prediction result of the test set, we randomly selected 1000 sentences with replacement for 100 times and generated 100 bootstrap data sets. For each bootstrap data set, we evaluated F-measures for three pairs of results: (1) “biLSTM + ELMo” and “biLSTM + ELMo + Flair,” (2) “biLSTM + ELMo + Flair” and “biLSTM + ELMo + Flair + semantic embedding,” and (3) “biLSTM + ELMo by Zhu et al [40]” and “biLSTM + ELMo + Flair + semantic embedding.” After that, we adopted a Wilcoxon signed rank test [58] to determine if the differences between F-measures from the three pairs were statistically significant. The results show that the improvement of F-measures for all three pairs were statistically significant (*P* values were .01, .02, and .03, respectively).

**Table 1:** Performance of all the models on the 2010 i2b2/VA dataset.

Model	F-1 (%)	Precision (%)	Recall (%)
Hidden semi-Markov^a^	85.23	86.88	83.64
LSTM^b^ by Liu et al [39]^a^	85.78	—^c^	—^c^
LSTM by Wu et al [43]^a^	85.94	85.33	86.56
BiLSTM^d^ + ELMo by Zhu et al [40]^a^	86.84 (0.16)	87.44 (0.27)	86.25 (0.26)
BiLSTM + Flair	87.01 (0.18)	87.54 (0.15)	86.49 (0.21)
BiLSTM + ELMo	87.01 (0.24)	87.64 (0.19)	86.40 (0.30)
BiLSTM + ELMo + Flair	87.30 (0.06)	87.78 (0.09)	86.85 (0.07)
BiLSTM + ELMo + Flair + semantic embedding	87.44 (0.07)	88.03 (0.14)	86.91 (0.10)

^a^Model is trained using the complete dataset of i2b2 2010, which contains 349 notes in the training set and 477 notes in the test set.

^b^LSTM: long short-term memory.

^c^Not reported.

^d^BiLSTM: bidirectional LSTM.

## Discussion

### Principal Findings

NER is a fundamental task in the clinical NLP domain. In this study, we investigated the effects of combinations of different types of embeddings on the NER task. We also explored how to use medical lexicon to further improve performance. Based on the result, we found that either ELMo or Flair embeddings could boost the system’s performance, and combining both embeddings could further improve the performance. Although both ELMo and Flair embeddings use biLM to train the LM on MIMIC-III corpus, they actually generate the contextualized word embeddings in different ways. ELMo concatenates all the biLM layers to represent all different levels of the knowledge, while Flair embedding is generated by a character-level LM. Character-level LM is different from character-aware LM [59] since it actually uses word-level LM while leveraging character-level features through a CNN encoding step. It was composed by the surrounding text’s embedding in the character-level. The difference between ELMo and Flair embeddings could explain the reason why they can play complementary roles in the model.

The results show that adding semantic embeddings could further improve performance. According to the study by Peters et al [35], the lower biLM layer specializes in local syntactic relationships, while the higher layers focus on modeling longer range relationships. Those relationships are learned from the pure clinical corpus without any resources from outside such as medical lexicons and ontologies. This study shows an effective way to incorporate domain knowledge into the deep neural network–based NER system.

A large amount of training data is required to achieve success when applying deep learning algorithms [60]. Within the general domain, it is more difficult to accumulate a large size of the annotated corpus for most of the clinical NLP tasks since it usually requires the annotator to have in-depth domain knowledge. Contextualized word embeddings, as an effective way of transferring the knowledge from the large unlabeled corpus, could address the issue of lack of training data. According to the results, by only using the small size of the training corpus (170 notes), contextualized word embedding–based models could achieve better performance than the models that use the large size training corpus (349 notes). To further investigate the effectiveness of transfer learning in our proposed models, we compared the performance of our best model generated from different sizes of the training data. Table 2 shows the F-1 score for the model “biLSTM + ELMo + Flair + semantic embedding” on randomly selected 80%, 60%, 40%, 20%, and 10% of the training data. Surprisingly, we found that using only 40% of the training corpus could achieve comparable performance as the original state-of-the-art traditional machine learning–based system. Even using 20% of the training corpus, the model’s F-1 score is still more than 80%. This result indicates that contextualized word representation could potentially be an effective way to reduce the size of the training corpus, which could significantly improve the feasibility of applying deep learning to real practice.

Besides the performance reported in the Results section, we also recorded the change of performance for our proposed models during the fine-tuning stage. Table 3 shows the F-1 score on 1, 20, 40, and 60 epochs for our three models. On epoch 1, comparing to only word embeddings, any contextualized word embedding boosts the F-1 score. This is mostly because pretraining on contextualized word embeddings is very beneficial for the task of named entity recognition. This proves that the LM is a good way for pretraining that can be adapted to different downstream NLP tasks. Another interesting finding is that even though the model ELMo achieved the best performance among our three models, it was surpassed by the other two models on later epochs, which indicates that during the optimization process, the best starting point does not necessarily lead to the best local optimal solution.

**Table 2:** Performance of the best model training, BiLSTM^a^ + ELMo + Flair + semantic embedding, on different sizes of the training corpus.

Amount of training data (%)	F-1 (%)	Prec (%)	Rec (%)
10	71.13	69.59	72.74
20	82.05	81.92	82.18
40	85.36	85.83	84.90
60	86.33	86.81	85.86
80	86.92	87.42	86.43

^a^BiLSTM: bidirectional long short-term memory.

**Table 3:** F-1 score for our proposed models on different epochs.

Model	1 epoch (%)	20 epochs (%)	40 epochs (%)	60 epochs (%)
Classic word embedding	61.23	75.67	78.11	79.52
Classic word embedding + ELMo	76.18	85.64	85.68	86.63
Classic word embedding + ELMo + Flair	73.28	85.33	85.97	86.96
Classic word embedding + ELMo + Flair + semantic embedding	74.38	85.85	86.46	87.13

### Limitations

This study has some limitations. For contextualized embedding generation, we followed others’ research methods and didn’t test different configurations for LM training. For example, for ELMo embeddings, we followed the work of Zhu et al [40] for Flair embedding generation and kept the same configuration as seen in the work by Akbik et al [36]. For the fine-tuning stage, we only fine-tuned a limited set of hyperparameters including learning rate and patience. For domain knowledge integration, there are a lot of options that could be explored to merge the lexicon information into the input of the deep neural network structure. In this study, we only tried one way to represent it in the form of word embeddings. In this paper, we studied two contextualized embeddings: ELMo and Flair. In the future, we plan to test our framework by adding bidirectional encoder representations from transformers, which is another popular contextualized embedding [61].

### Conclusions

In this study, we investigated the effects of the combination of two contextualized word embeddings including ELMo and Flair and clinical knowledge for the clinical NER task. Our evaluation on the 2010 i2b2/VA challenge dataset shows that using both ELMo and Flair embeddings outperforms using only ELMo embeddings, which indicates its great potential for the clinical NLP research. Furthermore, we demonstrate that incorporating the medical lexicon into the word representation could further improve the performance. Finally, we found that adopting our best model would be an effective way to reduce the size of the required training corpus for the clinical NER task.

## Abbreviations

biLM: bidirectional language model
biLSTM: bidirectional long short-term memory
CNN: convolutional neural network
CRF: conditional random field
HMM: hidden Markov model
i2b2: Informatics for Integrating Biology and the Bedside
LM: language model
LSTM: long short-term memory
MIMIC-III: Medical Information Mart for Intensive Care III
NER: named entity recognition
NLP: natural language processing
RNN: recurrent neural network
SSVM: structural support vector machine
SVM: support vector machine
VA: Veterans Affairs
